# Bayesian inference of environmental effects on seaweed production in Japan via a production-environmental suitability model

**DOI:** 10.1186/s40529-018-0250-x

**Published:** 2019-02-01

**Authors:** Hungyen Chen

**Affiliations:** Department of Agronomy, National Taiwan University, Number 1, Section 4, Roosevelt Road, Taipei, 10617 Taiwan

**Keywords:** Bayesian estimation, Climatic change, Seaweed growth, Yield prediction

## Abstract

**Background:**

Both natural and human-induced disturbances are commonly responsible for an overall decrease of the world’s seaweed. Along Japan’s coastal areas, edible seaweed production has been decreasing for decades. In this study, a production-environmental suitability model to estimate the impacts of environmental factors on seaweed production was developed. The developed model not only estimates human-induced disturbances but also quantifies the impacts of environmental factors responsible for the decline of annual seaweed production. The model estimated the temporal variation in human-induced disturbances and the effects of environmental factors (i.e., rainfall, CO_2_ concentrations, temperature, typhoons, solar radiation, water nutrient levels, and water quality) on edible seaweeds in Japan from 1985 to 2012.

**Results:**

The environmental suitability for seaweed production in Japan was about 4.6 times greater in 1992 than in 2011, meanwhile as a result of human activities, human-induced disturbances of seaweed increased at a rate of 4.9 times faster during the period of 1998–2012 than the period of 1985–1997. The ratio of decreased production to decreased environmental suitability for seaweed production in Japan increased by 15.2% during the study years, which means that seaweed production has become more sensitive to environmental disturbances, including climatic factors and human activities in 1998–2012.

**Conclusions:**

The results are novel in demonstrating temporal variations in the level of environmental suitability to seaweed production by using a simple mathematical model. The production-environmental suitability model successfully predicted seaweed production by reflecting the 28-year temporal variation of the observed seaweed production in Japan.

## Background

Seaweed, bathed in nutrient rich seawater, is a family of algae and is one of the most important aquatic living resources of the world’s oceans (Dhargalkar and Pereira 2005). Since 300 BC, seaweeds have been a source of food, feed, fertilizer, chemicals, medicine, and storm protection (Chapman 1970; Verkleij 1992; Smit 2004; Rönnbäck et al. 2007). The majority of the world’s seaweed cultivation, production, and consumption occurs particularly in East and Southeast Asian, e.g., China and Japan (Dhargalkar and Pereira 2005).

Seaweeds play a central ecological role in coastal habitats; as ecosystem engineers, they assist in supplying oxygen and absorbing CO_2_ to the sea (Hemminga and Duarte 2000; Graham 2004). Seaweeds act as one of the primary producers and competitors in the marine food chain (Graham 2004; Norderhaug et al. 2005), with some having the capacity to remove heavy metals from the seawater and potentially being used as bio-indicators for heavy metal pollution (Johansen et al. 1991; Phillips and Segar 1986; Campanella et al. 2001; Serfor-Armah et al. 2006) and can remove nutrients from the water column and inhibit eutrophication (Harlin and Thorne-Miller 1981). They also provide an important niche, e.g., create habitat for living, eating, parenting, and hiding, for plenty of marine species, and thus maintain community diversity (Stachowicz et al. 2008). However, although seaweed in general has good survival strategies, enabling the algae to withstand environmental stresses, the impacts of climatic change and human-induced disturbances on seaweeds remain poorly understood (Chan et al. 2006).

Seaweeds depend on an adequate degree of water quality and clarity to sustain productivity in their growth environment (Lüning 1990; Lobban and Harrison 1994). Seaweed loss, however, is increasing due to (1) natural disturbances, such as hurricanes, earthquakes, disease, and grazing by herbivores; and (2) human activities, including nutrient and sediment loading from runoff and sewage disposal, dredging and filling, pollution, upland development, and certain fishing practices (Sousa 1979; Chapman and Johnson 1990; Rainbow 1995; Short and Burdick 1996; Airoldi 1998; Coelho et al. 2000). The loss of seaweed influences the biodiversity of the ecosystem’s communities (Graham 2004; Norderhaug et al. 2005) and should be of concern to society.

Japan is over 3000 km long from the Okhotsk Sea to the Philippine Sea in the Pacific Ocean and has 29,751 km long coastline, the sixth longest in the world (MHLW 2018). As an island nation, Japan harvests numerous types of seaweed from all around the country (MAFF 2018). Seaweed farming began in Japan as early as 1670 in Tokyo Bay and seaweeds and seaweed-derived products have been central ingredients of Japanese cuisine for centuries (Borgese 1980). In 2012, Japan dedicated about 100,000 tonnes to seaweed production around the country (MAFF 2018).

Previous models for seaweed have used to simulate the growth and optimal annual harvesting strategy (Lee and Ang 1991), study the relationships between environmental factors and annual growth pattern (Friedlander et al. 1990), and explore the critical environmental parameters influencing the growth and establishment (Murphy et al. 2016). Cabral et al. (2016) proposed an approach to present an assessment of the potential impact of the installation of seaweed farms on ecosystem services and the induced compensation costs. Préat et al. (2018) used a method to model the seaweed production and develop indicators to assess the potential reductions in fisheries yield due to seaweed farming.

The crop yield-fertility model, modified from Michaelis–Menten kinetics (Michaelis and Menten 1913), was developed to estimate the soil fertility level and to quantify the contributions of fertilizer to the improvements in crop yields (Chen et al. 2014). It was used to interpret the effects of soil fertility and fertilizers on the crop yield and the extent of saturation of additional inputs. The model can be used to predict crop yields given the total fertility level of the soil and to describe the variation in crop yield and the effect of fertilizer treatment over years for crops using the estimated soil fertility. Bayesian inference of environmental effects on seaweed production.

In this study, a production-environmental suitability model, which was modified from the crop yield-fertility model, to estimate the effect of environmental suitability on seaweed production using Bayesian algorithm was developed. One of the major advantages of the Bayesian approach is to incorporate prior information (Box and Tiao 1973). The Bayesian approach forces the analyst to canvass expert knowledge by setting priors to determine what is known about the biological or environmental parameters and processes (Punt and Hilborn 1997). The developed model not only estimates human-induced disturbance but also quantifies the disturbance impacts of environmental factors (i.e., rainfall, CO_2_ concentrations, temperature, typhoons, solar radiation, water nutrient levels, and water quality) contributing to the decline of annual seaweed production in Japan. This paper defines human-induced disturbance as the combination of pollution, upland development, fishing practices, and other human activities that disrupt seaweed production. I used the model to estimate the annual variation in human-induced disturbances and the effects of environmental factors on edible seaweeds along the coastal areas around Japan from 1985 to 2012.

## Methods

### Production-environmental suitability model

Michaelis–Menten kinetics (Michaelis and Menten 1913) and the crop yield-fertility model (Chen et al. 2014) were taken and modified to produce this study’s seaweed model.

1 $$\begin{aligned} {\text{Y}}_{\text{t}} & \cong \frac{V}{{1 + \frac{K}{{ES_{t} }}}} \\ ES_{t} & = & \frac{1}{{HD_{t} }} + \frac{a}{{{\text{R}}_{\text{t}} }} + \frac{b}{{{\text{C}}_{\text{t}} }} + \frac{c}{{{\text{T}}_{\text{t}} }} + \frac{d}{{{\text{W}}_{\text{t}} }} + e \times {\text{S}}_{\text{t}} + f \times {\text{N}}_{\text{t}} + g \times {\text{Q}}_{\text{t}} \\ \end{aligned}$$

The model was used to interpret the effects of human-induced disturbances, rainfall, CO_2_ concentrations, temperature, typhoons, solar radiation, water nutrient levels, and water quality on seaweed production and the extent of saturation of additional inputs of environmental suitability for seaweed production. The model related productions (Y_t_) to the environmental suitability (*ES*_*t*_) of the seaweed at time *t*. *V* is the maximum production in response to the maximum environmental suitability; *K* is the suitability level before disturbances of human and environmental factors that is required to produce half of the maximum production. Human-induced disturbance (*HD*_*t*_) affecting the seaweed at time *t* is assumed to gradually vary over time. As previously mentioned, this paper defines human-induced disturbance as the combination of pollution, upland development, fishing practices, and other human activities. In other words, human-induced disturbance represents all the factors which relate to human activities and may influence the seaweed production. R_t_ is the value of rainfall at time t, C_t_ is the value of CO_2_ concentrations at time t, T_t_ is the value of sea surface temperature at time t, W_t_ is the value of number of typhoons at time t, S_t_ is the value of solar radiation at time t, N_t_ is the value of water nutrient levels at time t and Q_t_ is the value of water quality at time t. *a*, *b*, *c*, *d*, *e*, *f*, and *g* represent the effects of R_t_, C_t_, T_t_, W_t_, S_t_, N_t_, and Q_t_ relative to the seaweed production, respectively. The values of R_t_, C_t_, T_t_, W_t_, S_t_, N_t_, and Q_t_ were transferred to the relative value to the mean value (from 1985 to 2012). In the model, rainfall, CO_2_ concentrations, sea surface temperatures, and typhoons were assumed to be the factors of disturbance for seaweed production, decreasing the environmental suitability for seaweed production. On the other hand, solar radiation, water nutrient levels, and water quality increased the environmental suitability for seaweed.

### Likelihood and priors

A Bayesian framework was adopted for parameter estimation. Here, a gradual change in the human disturbance was assumed for analysis. However, we have to keep in mind that human disturbance is sometimes more often of abrupt or exponential change. The likelihood of the production at time *t* followed a normal distribution with the mean $\frac{V}{{1 + \frac{K}{{\frac{1}{{HD_{t} }} + \frac{a}{{{\text{R}}_{\text{t}} }} + \frac{b}{{{\text{C}}_{\text{t}} }} + \frac{c}{{{\text{T}}_{\text{t}} }} + \frac{d}{{{\text{W}}_{\text{t}} }} + e \times {\text{S}}_{\text{t}} + f \times {\text{N}}_{\text{t}} + g \times Q_{\text{t}} }}}}$ and the variance *δ*:

2 $${\text{Y}}_{\text{t}} \sim N\left( {\frac{V}{{1 + \frac{K}{{\frac{1}{{HD_{t} }} + \frac{a}{{{\text{R}}_{\text{t}} }} + \frac{b}{{{\text{C}}_{\text{t}} }} + \frac{c}{{{\text{T}}_{\text{t}} }} + \frac{d}{{{\text{W}}_{\text{t}} }} + e \times {\text{S}}_{\text{t}} + f \times {\text{N}}_{\text{t}} + g \times Q_{\text{t}} }}}},\delta } \right) .$$

Setting the HD in the first year to 1 normalized this value. The smoothness priors of the *HD* from the second year followed a normal distribution with the mean equal to the value of the *HD* in the preceding year and the variance *τ*:

3 $$HD_{t} \sim N\left( {HD_{t - 1} ,\tau } \right),\quad t > { 1}.$$

The inverse of *δ* followed a gamma distribution with a shape parameter of 0.1 and a scale parameter of 10. The inverse of *τ* followed a gamma distribution with a shape parameter of 10 and a scale parameter of 0.1. The prior of *V* followed a normal distribution with a mean of 0 and a standard deviation of 10^4^. The priors of *K*, *a*, *b*, *c*, *d*, *e*, *f*, and *g* followed a gamma distribution with a shape parameter of 1 and a scale parameter of 1. The priors of the estimates were designed to be as non-informative as possible within a realistic range of the parameter values. Runs of estimates in the model were performed using a Bayesian Markov chain Monte Carlo (MCMC) method as implemented in the software program WinBUGS (Lunn et al. 2000) and ran from R (R Development Core Team 2013) using the package R2WinBUGS (Sturtz et al. 2005). The number of iteration of the MCMC operation was set to 1,000,000 and thin was set to 100. All calculations and data analyses were performed using R v 3.0.2 (R Development Core Team 2013).

### Seaweed production in Japan

The data of yearly edible seaweed (i.e., kelp from the family Laminariaceae, *Undaria pinnatifida*, *Sargassum fusiforme,* and other edible seaweeds not harvested from aquiculture) production in the coastal areas around Japan from 1985 to 2012 were downloaded from the official website of the Ministry of Agriculture, Forestry and Fisheries (MAFF 2018). In this study, the fishery records were assumed to reflect the seaweed production. The size of growth area for seaweed can also respond to environmental change well, however it may be difficult to measure and evaluate annually for a long period around Japan, especially the records for the early years could be hard to obtain.

### Data of environmental factors

**Fig. 1:**
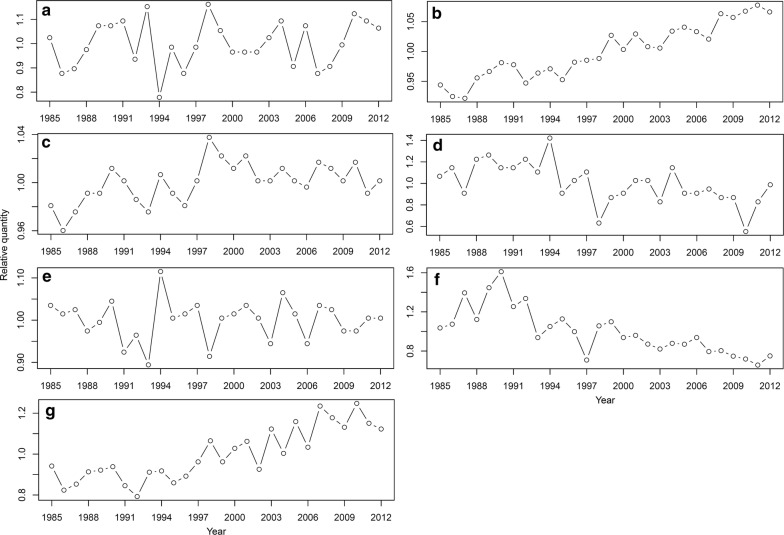
Temporal variation of the environmental factors in Japan from 1985 to 2012. **a** Rainfall. **b** Sea water CO_2_ concentration. **c** Sea surface temperature. **d** Number of typhoon. **e** Solar radiation. **f** Sea water nutrient level. **g** Sea water quality. The value in each year was transferred to the ratio of the mean value among the 28 years for each environmental factor. See “Methods” for details

Annual data for precipitation, CO_2_ concentrations in the seawater, sea surface temperatures, number of typhoons, and solar radiation in Japan were downloaded from the Japan Meteorological Agency official website (available online from http://www.jma.go.jp/jma/menu/menureport.html). Data for precipitation was collected using annual values from around Japan. To measure CO_2_ concentrations, data were collected using values along coastal areas of central Japan (137ºE, 34ºN) in winter. Sea surface temperatures were collected using the annual mean in the seas around Japan. The number of recorded typhoons was based on the number of occurrences around Japan. Solar radiation data was based on the annual value of sunlight duration around Japan. Seawater nutrient levels and seawater quality were downloaded from the Japanese Ministry of Land, Infrastructure, Transport and Tourism official website (available online from http://www.pa.cgr.mlit.go.jp/chiki/suishitu/). The data for seawater nutrient levels and seawater quality were collected using the annual value in the Seto Inland Sea in Japan, with nutrient levels calculated as the sum of concentrations of discharged loads of total phosphorus (TP), total nitrogen (TN), dissolved inorganic phosphate (PO_4_), and nitrogen (NO_3_, NO_2_ and NH_4_). Seawater quality was assessed according to water transparency values. To integrate the collected data, the value in each year was transferred to the ratio of the mean value among the 28 years for each environmental factor (Fig. 1). The mean values during the growth season of environmental parameters are reasonable for the analyses of growth in plants and algae. The annual means were used here because this analysis included different kinds of seaweeds at large-ranged areas (latitude) with variable suitable growth seasons and it may be difficult to select an appropriate period for growth season. Although annual mean may not reflect the exact situation for the seaweed growth, it could reflect the annual variation among different years in long-term temporal analyses.

## Results

### Decreasing trend of seaweed production

**Fig. 2:**
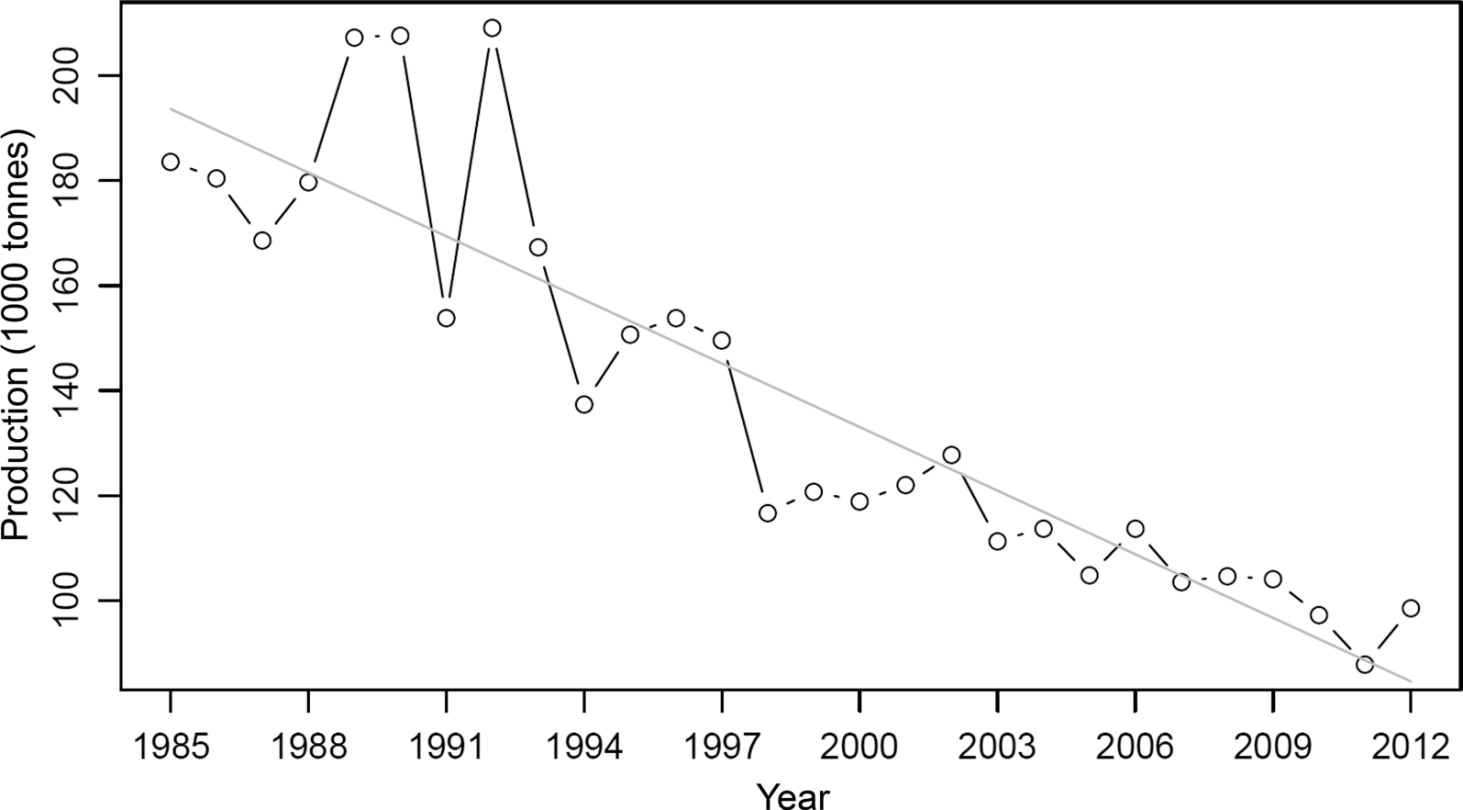
Temporal variations in observed seaweed production (1000 tonnes) in Japan from 1985 to 2012. The gray line represents the simple linear regression line

According to Fig. 2, the annual temporal variation in seaweed production in Japan ranged between 87,779 (in 2011) and 209,141 (in 1992) tonnes with a mean of 139,119 and standard deviation 36,808 during the 28 years from 1985 to 2012. A decreasing trend of temporal variation was found by using simple linear regression of the production in response to the year during 1985–2012 (slope =  − 039; *p* value < 0.001).

### Maximum production and environmental suitability

**Fig. 3:**
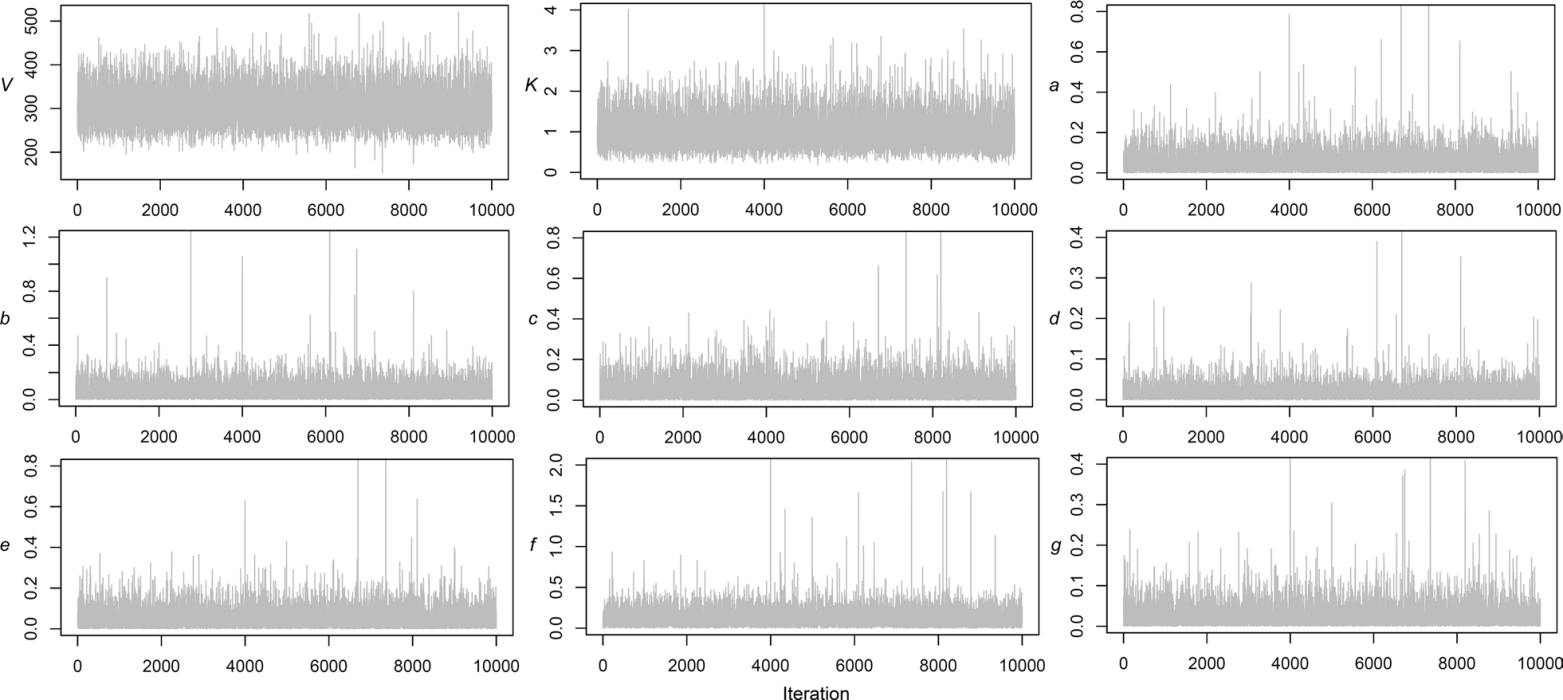
Traces of the MCMC samples of *V* (1000 tonnes), *K*, *a*, *b*, *c*, *d*, *e*, *f* and *g*. The chain length was set to 1,000,000 steps logging every 100th step. *V*, maximum production; *K*, half-saturated suitability; *a*, effect of rainfall; *b*, effect of sea water CO_2_ concentration; *c*, effect of temperature; *d*, effect of typhoon; *e*, effect of solar radiation; *f*, effect of sea water nutrient level; *g*, effect of sea water quality

Table 1 shows the results of the Bayesian estimates (*V*, *K*, *a*, *b*, *c*, *d*, *e*, *f*, and *g*) of the model for seaweed production in Japan. The traces of the MCMC samples show good mixing and convergence to the posterior distributions (Fig. 3). The largest observed seaweed production in Japan for 1 year was 209,141 (1992), which is 68.7% of the estimated maximum production (*V*, 304,508 tonnes). The environmental suitability (*ES*_*t*_) for each year was estimated by using environmental variables (R_t_, C_t_, T_t_, W_t_, S_t_, N_t_, and Q_t_) given the estimates *HD*_*t*_, *a*, *b*, *c*, *d*, *e*, *f*, and *g*. The estimated environmental suitability for seaweed in Japan had a mean of 0.925 and standard deviation of 0.518 during the 28 years. The estimated environmental suitability ranged between 0.459 (in 2011) and 2.148 (in 1992), which are 0.46 × half-saturated suitability (*K*, 1.008) and 2.13 × half-saturated suitability for seaweed in Japan. This means that the environmental suitability for seaweed in Japan decreased from more than 2 times (2.13 × *K*) to about half of half-saturated suitability (0.46 × *K*) over the years. Therefore, the environmental suitability for seaweed production in Japan was about 4.6 times (2.13/0.46) greater in 1992 than in 2011. The environmental suitability was first estimated below half-saturated suitability in 1994 (0.833) and has never reached half-saturated suitability again since then.

**Table 1:** The posterior mean and standard deviation (SD) of the Bayesian estimates

Factor	Estimate	Mean	SD	Percentage of contribution to all the environmental factors (%)
Maximum production (1000 tonnes)	*V*	304.508	43.085	–
Half-saturated suitability	*K*	1.008	0.421	–
Rainfall	*a*	0.054	0.053	13.2
CO_2_ concentration	*b*	0.065	0.072	15.9
Temperature	*c*	0.055	0.065	13.5
Typhoon	*d*	0.018	0.022	4.4
Solar radiation	*e*	0.053	0.060	13.0
Nutrient level	*f*	0.137	0.119	33.6
Water quality	*g*	0.026	0.039	6.4

### Effects of environmental factors

The Bayesian estimates for the contribution of environmental factors to seaweed and their percentages of contribution in the studied period are showed in Table 1. The impact from nutrient levels (*f*, 0.137; 33.6%) was the largest among the seven environmental factors; the second largest was CO_2_ concentrations (*b*, 0.065; 15.9%); third was temperature (*c*, 0.055; 13.5%); fourth was rainfall (*a*, 0.054; 13.2%); solar radiation (*e*, 0.053; 13.0%) was fifth; second last was water quality (*g*, 0.026; 6.4%); and last was number of typhoons (*d*, 0.018; 4.4%). This result suggests that nutrient levels may play the most influential role, of the seven environmental factors, regarding seaweed production in Japan. In fact, the impact was twice as large as seawater CO_2_ concentrations, the second largest impact. Temperature, rainfall, and solar radiation impacts were all similar in value. Water quality had the smallest impact among the selected environmental factors, which had an effect of about one-fifth the significance of seawater nutrient levels.

### Increasing trend of human-induced disturbance

**Fig. 4:**
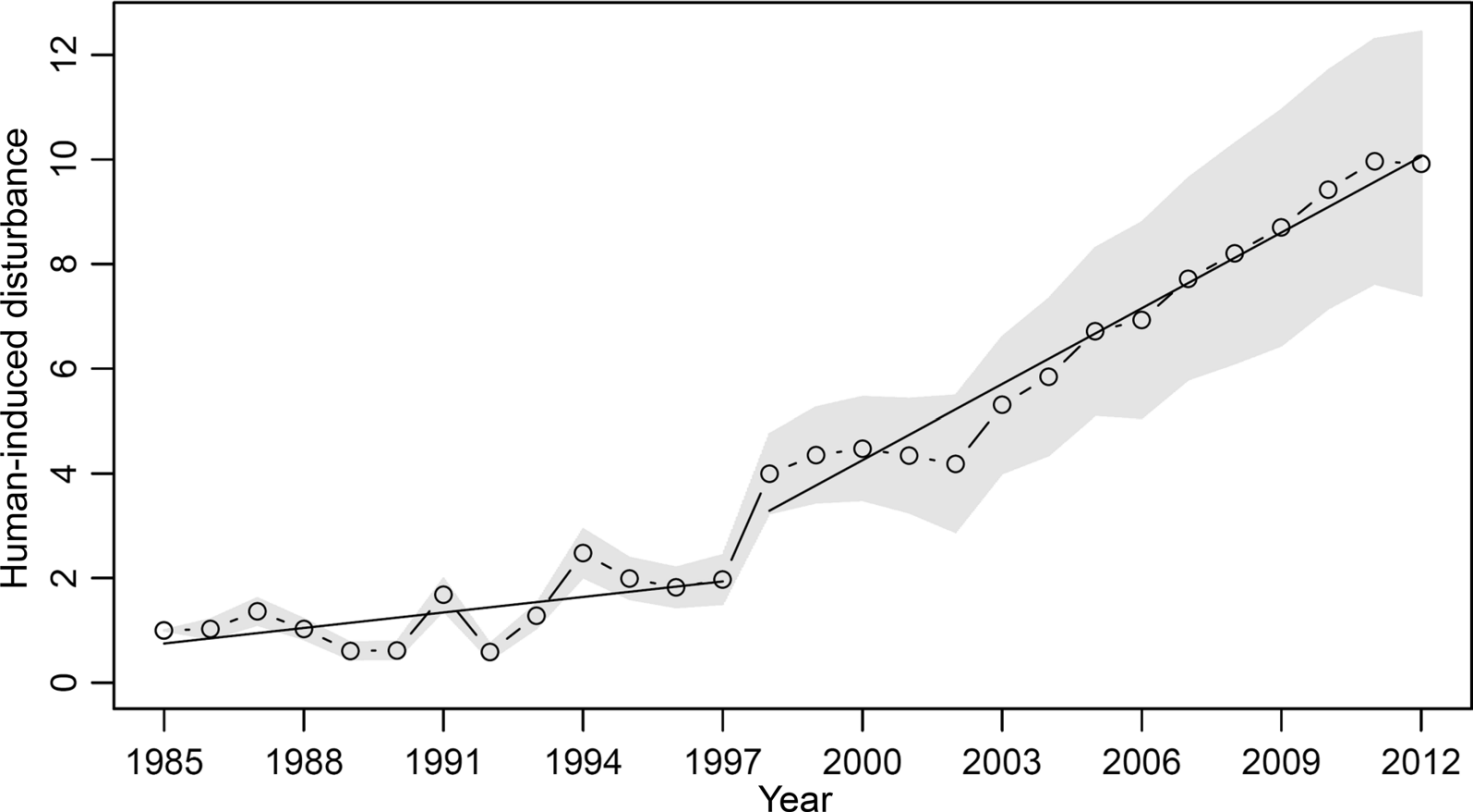
Temporal variations in the posterior mean and the gray band that corresponds to the standard deviation (± SD) of the Bayesian estimates of human-induced disturbance to seaweed in Japan from 1985 to 2012. The straight lines represent the simple linear regression lines. The value in the initial year was set to 1

The estimated human-induced disturbance (*HD*_*t*_) to seaweed in Japan was highly correlated with year (correlation coefficient = 0.957; p-value < 0.001) and has been increasing over the 28-year period (slope of simple linear regression = 0.368; p-value < 0.001). Setting the initial year (1985) to 1 normalized the value of human-induced disturbance. Figure 4 shows the long-term temporal variation of the human-induced disturbance to seaweed and its standard deviation band in Japan.

The trend of human-induced disturbance has been increasing since 1998 (Fig. 4). The correlation coefficient for human-induced disturbance to year was 0.636 (p-value = 0.02), and slope of simple linear regression was 0.099 (p-value = 0.02) during 1985–1997 (Fig. 4). However, the slope of simple linear regression became 0.483 (p-value < 0.001) with a correlation coefficient, 0.980 (p-value < 0.001) during 1998–2012 (Fig. 4). The results suggest a much faster rate of increase of the human-induced disturbance to seaweed in Japan during the period of 1998–2012 than the period of 1985–1997 (about 4.9 times faster).

### Predicted crop yields reflected the trend of the observed yields

**Fig. 5:**
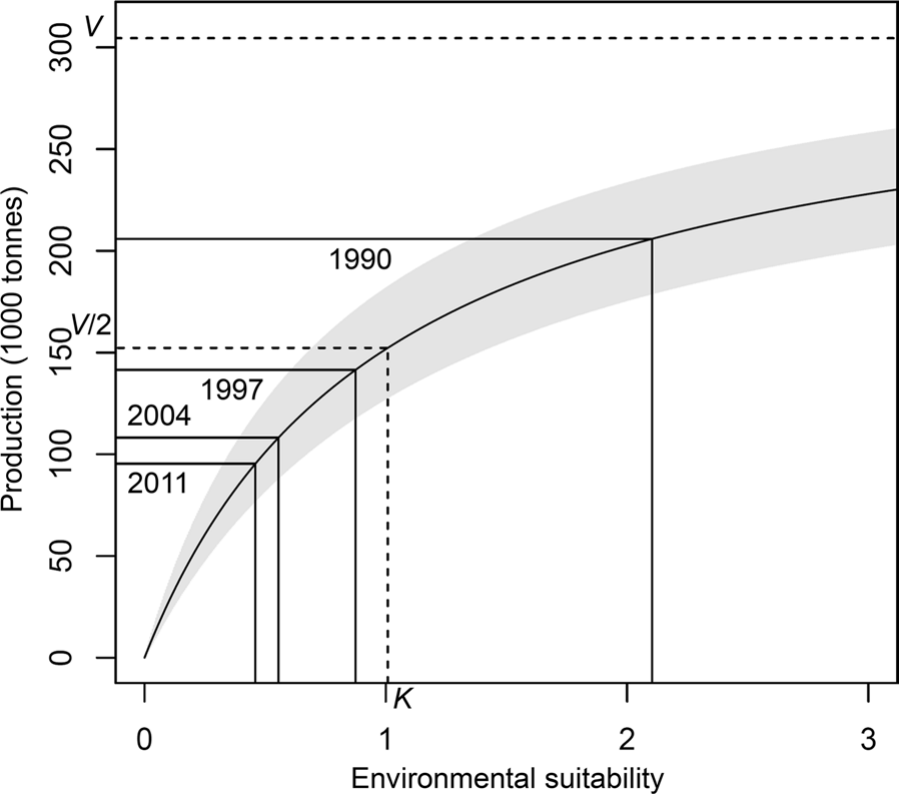
Production-environmental suitability curve for seaweed in Japan. The gray band represents the band that corresponds to the standard deviation (± 0.5 SD) of the curve. *V*, maximum production; *K*, half-saturated suitability

**Fig. 6:**
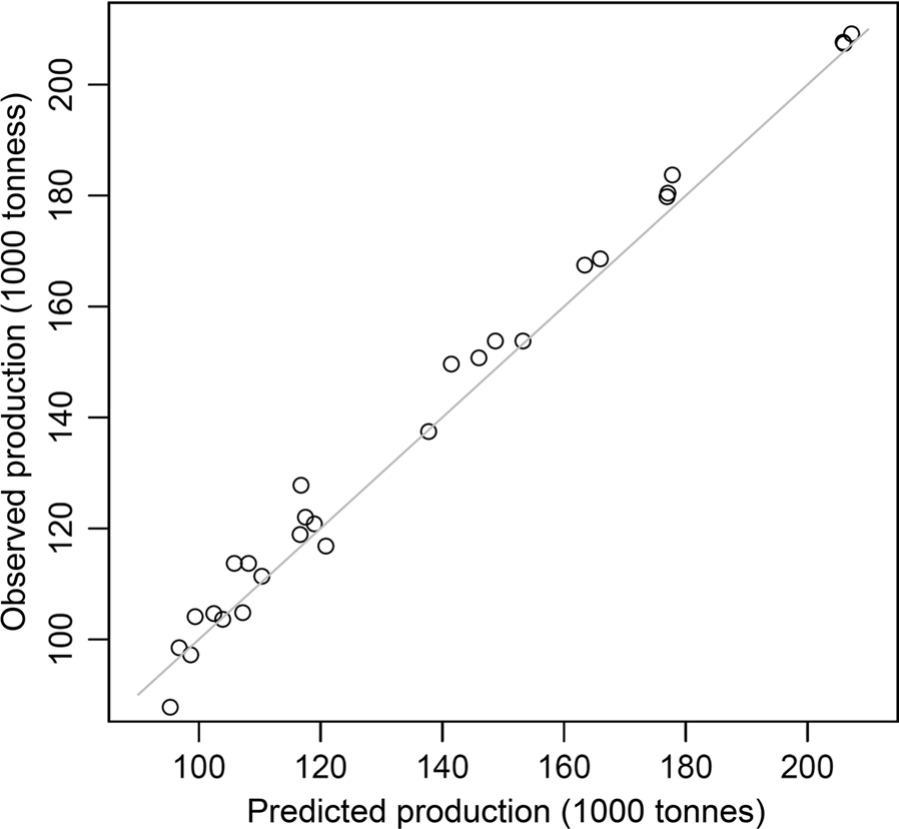
The relationship between the observed and predicted seaweed productions in Japan. The gray line represents diagonal 1:1 line

Using the estimated maximum production, half-saturated suitability and environmental suitability, the range of predicted production was estimated by the production-environmental suitability model for seaweed in Japan from 1985 to 2012. The estimated environmental suitability was transformed to the seaweed production by the production-environmental suitability curve (Fig. 5). The predicted seaweed production had a range of 95,321–207,241 tonnes with a mean of 136,666 and standard deviation of 36,031 during the 28 years. The predicted production was highly correlated with the observed production for seaweed in Japan (correlation coefficient = 0.995; p-value < 0.001; Fig. 6).

The environmental suitability was estimated at 2.103 in 1990, 0.875 in 1997, 0.555 in 2004, and 0.459 in 2011, yielding 205,857, 141,475, 108,156, and 95,321 tonnes of seaweed, respectively (Fig. 5). The environmental suitability decreased by 1.229, then 0.319, and then 0.096 in these three 7-year periods (1990–1997, 1997–2004, 2004–2011), respectively, which resulted in an overall decrease of 64,382, 33,319, and 12,835 tonnes of seaweed production, respectively (Table 2). Compared with the value in the initial year of each period, the environmental suitability decreased by 58.4%, 36.5%, and 17.3%, respectively, resulting in a decrease in seaweed production by 31.3%, 23.6%, and 11.9%, respectively (Table 2). The ratios of the decreased production to the decreased environmental suitability were 53.5%, 64.5%, and 68.7% for these three periods, respectively (Table 2). The ratio of the decreased production to the decreased environmental suitability for the seaweed production in Japan increased by 15.2% (68.7%–53.5%) during the years. Figure 5 shows the increasing ratio of the decreased production to the decreased environmental suitability. This result suggests that in 1998–2012, seaweed production in Japan has become more and more sensitive to environmental disturbances, including climatic factors and human activities.

**Table 2:** Environmental suitability and production for seaweed in Japan estimated using the production-environmental suitability model

Year	Environmental suitability			Production			Ratio of decreased percentage of production to decreased percentage of environmental suitability (%)
	Estimated value	Decreased value	Decreased percentage (%)	Estimated value (ton)	Decreased value (ton)	Decreased percentage (%)	
1990	2.103	–	–	205,857	–	–	–
1997	0.875	1.229	58.4	141,475	64,382	31.3	53.5
2004	0.555	0.319	36.5	108,156	33,319	23.6	64.5
2011	0.459	0.096	17.3	95,321	12,835	11.9	68.7

See the production-environmental suitability curve in Fig. 5

## Discussion

Both large-scale and local losses of seaweed have been frequently reported around the world for decades (Short and Burdick 1996; Coelho et al. 2000; Heck et al. 2003; Orth et al. 2006; Airoldi and Beck 2007). Natural events, such as extreme climatic events, have been identified as causes of seaweed loss in temperate and tropical regions (Orth et al. 2006). Reported anthropogenic threats to seaweed health include global warming, shifts in water quality, and increased loading of sediment, contaminants, and nutrients (Kemp et al. 2005; Orth et al. 2006; Walker et al. 2006). Extreme climatic events (e.g., frequency and intensity of storms, hurricanes, typhoons, and associated surges and swells) also can have large-scale impacts on seaweed (Orth et al. 2006). However, Short and Wyllie-Echeverria’s (1996) findings suggest that, although natural events have been responsible for loss of seaweed habitat, the most serious cause of seaweed loss is attributable to human population expansion and increasing anthropogenic inputs to the coastal oceans.

The environmental factors analyzed in the study were selected because: the amount of solar radiation are related to seaweed communities (Bischof et al. 2006); the degrees of water temperature and CO_2_ concentrations are positively correlated and they reflect the degrees of global warming and ocean acidification which affect seaweed communities (Pörtner 2008; Harley et al. 2012); the amount of precipitation and the number of typhoons can be considered as the degree of environmental disturbances (Short and Wyllie-Echeverria 1996); water nutrient levels and water quality are related to the growth of seaweed (Msuya and Neori 2008). Among these selected environmental factors, the results suggest nutrient levels in seawater may have the largest impact on seaweed production in Japan. Various nutrients in different concentrations come from industrial pollutants, agricultural runoff, municipal sewage systems, and other human activities. Seawater CO_2_ concentrations are also significant environmental factors, suggesting that pH value could be another important factor in the production of seaweed in Japan and could also be correlated with the human activities.

Generally, the reasons behind the global reduction of seaweed could be due to changing sea currents, sea temperatures, nutrient levels, and the abundance of seaweed-eating species, such as fish, shellfish, and sea urchins (Burkepile and Hay 2006; Filbee-dexter and Scheibling 2014; Ling et al. 2014). At the local scale, the loss of seaweed could be due to the varied coastal topography, seafloors, seawater transparency, and eutrophication (Lüning 1990; Schramm 1999; Walker et al. 2006; Yang et al. 2015). The loss of seaweed has been a serious problem in Japan since early 2000s (Fujita et al. 2006; Fujita 2009). Besides the reasons mentioned above, these losses are most likely due to a combination of many natural factors such as increased sea surface temperatures, decreased nutrient levels, consumption by herbivores, inflow of fresh water from rivers, extreme climatic events, and human-induced factors such as coastal destruction, industrial and agricultural pollution, drainage from power plants, fisheries, aquaculture, and overfishing (Nakatsu 2005; Fujita 2009; Yamaguchi 2010; Fisheries Agency 2015). The increasing rate of human-induced disturbance to seaweed production suggested by the results of this study may be due to a high increase in human activities such as, pollution, upland development, and fishing practices (Short and Wyllie-Echeverria 1996; Dulvy et al. 2003; Crain et al. 2009). The other serious problem in some of the shallow seas along the Japanese coast have been suffering from a phenomenon known as barren ground, sea desert or denudation of rocks (Fujita 2010). This phenomenon was known and observed in Japan, however it may be difficult to measure and evaluate annually for a long period and a large-scaled area.

The objectives of this study are to estimate the human-induced disturbance and to quantify the disturbance impacts of environmental factors contributing to the decline of annual seaweed loss in Japan by using a mathematical model. However, one point concerns the temporal data of seaweed production may not reflect the exact temporal variation of seaweed abundance. The quality and reliability of this analysis could be improved by including the information of the efforts involved to get the seaweed production, market demand of seaweed, and the number of fisherman every year. Because it is very difficult to get this information for the data of large-scaled investigation in several years, we may assume these variables were consistent during the studying period. We have to keep this point in mind and interpret the results carefully. The model used in this study can be applied at other areas in the world, as long as the information of environmental variables is provided. In addition, the model can be revised easily depending on what kind of information or type of data we have.

## Conclusions

By assuming that the fishery records can reflect the seaweed production, the results succeeded in demonstrating a mathematical method to estimate the increasing effect of human-induced disturbance to the seaweed community. The environmental suitability for seaweed in Japan was about 4 times greater in 1992 than in 2011. The proposed model was used to estimate the size of the effects of different environmental factors. The results show that human activity related factors, i.e., seawater nutrient levels and CO_2_ concentrations, had larger impacts on the variation of seaweed production in Japan. Human-induced disturbance of seaweed increased at a rate 4.9 times faster during the period of 1998–2012 than the period of 1985–1997. The ratio of the decreased production to the decreased environmental suitability for seaweed production in Japan increased by 15.2% during the years, which means that the seaweed production has become more sensitive to environmental disturbances in 1998–2012, including climatic factors and human activities. The results are novel in demonstrating temporal variations in the level of environmental suitability to seaweed production by using a simple mathematical model. The production-environmental suitability model successfully predicted seaweed production by reflecting the 28-year temporal variation of the observed seaweed production in Japan. Thereby this study has provided a method to estimate the effects of human-induced disturbances and climatic changes on aquatic plants.

## Abbreviations

ES: environmental suitability
HD: human-induced disturbance

## References

[CR1] Agency Fisheries (2015). Isoyake Taisaku Guideline Re-ed.

[CR2] Airoldi L (1998). Roles of disturbance, sediment stress, and substratum retention on spatial dominance in algal turf. Ecology.

[CR3] Airoldi L, Beck MW (2007). Loss, status and trends for coastal marine habitats of Europe. Oceanogr Mar Biol Annu Rev.

[CR4] Bischof K, Gómez I, Molis M, Hanelt D, Karsten U, Lüder U, Roleda MY, Zacher K, Wiencke C (2006). Ultraviolet radiation shapes seaweed communities. Rev Environ Sci Bio.

[CR5] Borgese EM (1980). Seafarm: the story of aquaculture.

[CR6] Box GEP, Tiao GC (1973). Bayesian inference in statistical analysis.

[CR7] Burkepile DE, Hay ME (2006). Hervibore vs. nutrient control of marine primary producers: context-dependent effects. Ecology.

[CR8] Cabral P, Levrel H, Viard F, Frangoudes K, Girard S, Scemama P (2016). Ecosystem services assessment and compensation costs for installing seaweed farms. Mar Policy.

[CR9] Campanella L, Conti ME, Cubadda F, Sucapane C (2001). Trace metals in seagrass, algae and mollusks from an uncontaminated area in the Mediterranean. Environ Pollut.

[CR10] Chan C-X, Ho C-L, Phang S-M (2006). Trends in seaweed research. Trends Plant Sci.

[CR11] Chapman VJ (1970). Seaweeds and their uses.

[CR12] Chapman ARO, Johnson CR (1990). Disturbance and organization of macroalgal assemblages in the Northwestern Atlantic. Hydrobiologia.

[CR13] Chen H, Yamagishi J, Kishino H (2014). Bayesian inference of baseline fertility and treatment effects via a crop yield-fertility model. PLoS ONE.

[CR14] Coelho SM, Rijstenbil JW, Brown MT (2000). Impacts of anthropogenic stress on the early development stages of seaweeds. J Aquat Ecosys Stress Recov.

[CR15] Crain CM, Halpern BS, Beck MW (2009). Understanding and managing human threats to the coastal marine environment. The year in ecology and conservation biology, 2009. Ann NY Acad Sci.

[CR16] Dhargalkar VK, Pereira N (2005). Seaweed: promising plant of the millennium. Sci Cul.

[CR17] Dulvy NK, Sadovy Y, Reynolds JD (2003). Extinction vulnerability in marine populations. Fish Fish.

[CR18] Filbee-dexter K, Scheibling RE (2014). Sea urchin barrens as alternative stable states of collapsed kelp ecosystems. Mar Ecol Prog Ser.

[CR19] Friedlander M, Galai N, Farbstein H (1990). A model of seaweed growth in an outdoor culture in Israel. Hydrobiologia.

[CR20] Fujita D (2009) Decline and restoration of seaweed beds. Tokyo Fisheries Promotion Foundation

[CR21] Fujita D (2010). Current status and problems of Isoyake in Japan. Bull Fish Res Agen.

[CR22] Fujita D, Ishikawa T, Kodama S, Kato Y, Notoya M (2006). Distribution and recent reduction of Gelidium beds in Toyama Bay, Japan. J Appl Phycol.

[CR23] Graham MH (2004). Effects of local deforestation on the diversity and structure of southern California giant kelp forest food webs. Ecosystems.

[CR24] Harley CDG, Anderson KM, Demes KW, Jorve JP, Kordas RL, Coyle TA, Graham MH (2012). Effects of climate change on global seaweed communities. J Phycol.

[CR25] Harlin MM, Thorne-Miller B (1981). Nutrient enrichment of seagrass beds in a Rhode Island coastal lagoon. Mar Biol.

[CR26] Heck KL, Hays G, Orth RJ (2003). Critical evaluation of the nursery role hypothesis for seagrass meadows. Mar Ecol Prog Ser.

[CR27] Hemminga M, Duarte CM (2000). Seagrass Ecology.

[CR28] Johansen P, Hansen MM, Asmund G, Nielsen PB (1991). Marine organisms as indicators of heavy metal pollution—experience from 16 years of monitoring at a lead zinc mine in Greenland. Chem Ecol.

[CR29] Kemp WM, Boynton WR, Adolf JE, Boesch DF, Boicourt WC, Brush G, Cornwell JC, Fisher TR, Glibert PM, Hagy JD, Harding LW, Houde ED, Kimmel DG, Miller WD, Newell RIE, Roman MR, Smith EM, Stevenson JC (2005). Eutrophication of Chesapeake Bay: historical trends and ecological interactions. Mar Ecol Progr Ser.

[CR30] Lee CS, Ang P (1991). A simple model for seaweed growth and optimal harvesting strategy. Ecol Model.

[CR31] Ling SD, Scheibling RE, Rassweiler A, Johnson CR, Shears N, Connell SD, Salomon AK, Norderhaug KM, Pérez-Matus A, Hernández JC, Clemente S, Blamey LK, Hereu B, Ballesteros E, Sala E, Garrabou J, Cebrian E, Zabala M, Fujita D, Johnson JE (2014). Global regime shift dynamics of catastrophic sea urchin overgrazing. Phil Trans R Soc B.

[CR32] Lobban CS, Harrison PJ (1994). Seaweed ecology and physiology.

[CR33] Lüning K (1990). Seaweeds, their environment, biogeography and ecophysiology.

[CR34] Lunn DJ, Thomas A, Best N, Spiegelhalter D (2000). WinBUGS—a Bayesian modelling framework: concepts, structure, and extensibility. Stat Comput.

[CR35] MAFF (2018) Ministry of Agriculture, Forestry and Fisheries. http://www.maff.go.jp/j/tokei/index.html

[CR36] MHLW (2018) Water Supply in Japan. Ministry of Health, Labour and Welfare. https://www.mhlw.go.jp/english/policy/health/water_supply/1.html

[CR37] Michaelis L, Menten ML (1913). Die kinetik der invertinwirkung. Biochem Z.

[CR38] Msuya FE, Neori A (2008). Effect of water aeration and nutrient load level on biomass yield, N uptake and protein content of the seaweed Ulva lactuca cultured in seawater tanks. J Appl Phycol.

[CR39] Murphy JT, Johnson MP, Viard F (2016). A modelling approach to explore the critical environmental parameters influencing the growth and establishment of the invasive seaweed *Undaria pinnatifida* in Europe. J Theor Biol.

[CR40] Nakatsu T (2005). Promotion of seaweed bed creation in fisheries infrastructure improvement. Fish Engine.

[CR41] Norderhaug KN, Christie H, Fossa JH, Fredriksen S (2005). Fish-macrofauna interactions in a kelp (*Laminaria hyperborea*) forest. J Mar Biol Assoc UK.

[CR42] Orth RJ, Carruthers TJB, Dennison WC, Duarte CM, Fourqurean JW, Heck KL, Hughes AR, Kendrick GA, Kenworthy WJ, Olyarnik S, Short FT, Waycott M, Williams SL (2006). A global crisis for seagrass ecosystems. Bioscience.

[CR43] Phillips DJH, Segar DA (1986). Use of bio-indicators in monitoring conservative contaminants: programme design imperatives. Mar Pollut Bull.

[CR44] Pörtner H-O (2008). Ecosystem effects of ocean acidification in times of ocean warming: a physiologist’s view. Mar Ecol Progr Ser.

[CR45] Préat N, De Troch M, van Leeuwen S, Taelman SE, De Meester S, Allais F, Dewulf J (2018). Development of potential yield loss indicators to assess the effect of seaweed farming on fish landings. Algal Res.

[CR46] Punt AE, Hilborn R (1997). Fisheries stock assessment and decision analysis: the Bayesian approach. Rev Fish Biol Fish.

[CR47] Rainbow PS (1995). Biomonitoring of heavy metal availability in the marine environment. Mar Pollut Bull.

[CR57] R Core Team (2013) R: A Language and Environment for Statistical Computing. R Foundation for Statistical Computing, Vienna, Austria http://www.R-project.org/

[CR48] Rönnbäck P, Kautsky N, Pihl L, Troell M, Söderqvist T, Wennhage H (2007). Ecosystem goods and services from Swedish coastal habitats: identification, valuation, and implications of ecosystem shifts. Ambio.

[CR49] Schramm W (1999). Factors influencing seaweed responses to eutrophication: some results from EU-project EUMAC. J Appl Phycol.

[CR50] Serfor-Armah Y, Carboo D, Akuamoah RK, Chatt A (2006). Determination of selected elements in red, brown and green seaweed species for monitoring pollution in the coastal environment of Ghana. J Radioanal Nuclear Chem.

[CR51] Short FT, Burdick DM (1996). Quantifying seagrass habitat loss in relation to housing development and nitrogen loading in Waquoit Bay, Massachusetts. Estuaries.

[CR52] Short FT, Wyllie-Echeverria S (1996). Natural and human-induced disturbance of seagrasses. Environ Conser.

[CR53] Smit AJ (2004). Medicinal and pharmaceutical uses of seaweed natural products: a review. J Appl Phycol.

[CR54] Sousa WP (1979). Experimental investigation of disturbance and ecological succession in a rocky intertidal algal community. Ecol Monogr.

[CR55] Stachowicz JJ, Graham M, Bracken MES, Szoboszlai AI (2008). Diversity enhances cover and stability of seaweed assemblages: the role of heterogeneity and time. Ecology.

[CR56] Sturtz S, Ligges U, Gelman A (2005). R2WinBUGS: a package for running WinBUGS from R. J Stat Softw.

[CR58] Verkleij FN (1992). Seaweed extracts in agriculture and horticulture: a review. Biol Agric Hortic.

[CR59] Walker DI, Kendrick GA, McComb AJ (2006) Decline and recovery of seagrass ecosystems—the dynamics of change. Orth RJ, Duarte CM, eds. Seagrasses: Biology, Ecology and Conservation. Dordrecht (The Netherlands). Springer

[CR60] Yamaguchi A (2010). Biological aspects of herbivorous fishes in the coastal areas of western Japan. Bull Fish Res Agen.

[CR61] Yang Y, Chai Z, Wang Q, Chen W, He Z, Jiang S (2015). Cultivation of seaweed Gracilaria in Chinese coastal waters and its contribution to environmental improvements. Algal Res.

